# First European Case of Serotype A *MAT****a*** *Cryptococcus neoformans* Infection

**DOI:** 10.3201/eid0909.020770

**Published:** 2003-09

**Authors:** M. A. Viviani, R. Nikolova, M.C. Esposto, G. Prinz, M.Cogliati

**Affiliations:** * Institute of Hygiene and Preventive Medicine, Università degli Studi di Milano, Milan, Italy; † St. Laszlo Hospital, Budapest, Hungary

**To the Editor**: *Cryptococcus neoformans* is an opportunistic fungus that causes meningoencephalitis, primarily in immunocompromised patients. However, *C. neoformans* can also cause illness in apparently normal hosts. The yeast is a heterothallic basidiomycete with two mating types, *MAT****a*** and *MATα* identified in all the four serotypes, A, B, C, and D. However, the mating type ***a*** of serotype A is a rare and recent finding. One strain was isolated from a Tanzanian AIDS patient and a second from the Italian environment; the first was mating defective (*1*,*2*). We report the isolation of a serotype A *MAT****a*** strain of clinical origin that was characterized by mating at high frequency under standard laboratory conditions.

In August 1998, a 45-year-old Hungarian man was admitted to the Laszlo Hospital for Infectious Diseases in Budapest because of septic fever. The patient had a history of hematologic malignancy (Hodgkin disease), which was diagnosed in 1991. He had received several courses of chemotherapy and radiation. After 4 years when his cancer was in remission, in September 1995, the disease recurred (stage IVa) for which he received several more courses of chemotherapy, according to protocols BEAM (carmustine, etoposide, cytarabine, melphalan) and CEP (lomustine, etoposide, prednimustine). In February 1998, another relapse was diagnosed and the patient was given chemotherapy, according to protocol COPP (cyclophosphamide, vincristine, prednisolone, procarbazine) four times. In April 1998, he was hospitalized with herpes zoster infection and treated with acyclovir. At the last admission, in August 1998, he was pancytopenic and had septic fever. *Salmonella enteritidis* was cultured from his blood. The salmonella septicemia was successfully treated with ceftriaxone. As palliative treatment, he received 4x10 mg vinblastine for his residual disease. On September 30, he became febrile again. *Cryptococcus neoformans* was isolated from his blood, although cerebrospinal fluid culture and serologic tests were negative. On the right fossa cubitalis, cellulitis and a tender mass were present, although he did not have a history of recent central line or cytostatic treatment on this side. *Cryptococcus neoformans* was isolated from the sample taken from the mass. Antifungal treatment was started with 600 mg fluconazole per day and continued with amphotericin B, 1 mg/kg/day. The patient died 6 weeks after the isolation of *Cryptococcus*, probably because of his uncontrolled Hodgkin disease. As far as the physician was aware, the patient had not visited other countries.

The strain, isolated from the patient’s blood during the European Confederation of Medical Mycology Cryptococcosis Survey, was sent for typing to the European Convenor. The isolate, IUM 99-3617, was identified as serotype A using Crypto Check serotyping kit from Iatron Laboratories (Tokyo, Japan) and genotyped as VN6 by multiplex polymerase chain reaction (PCR) (*3*) by using the primers previously described (*4*,*5*). The fungus was shown to be haploid by cytofluorimetric analysis (*6*). The strain’s fertility was investigated, according to Kwon-Chung (*7*), by crossing the isolate with reference serotype A strains H99 (*MATα*) and IUM 96-2828 (*MAT****a***), and with serotype D congenic strains JEC20 (*MAT****a***) and JEC21 (*MATα*). When cocultured with *MATα* strains (H99 and JEC21), IUM 99-3617 produced abundant basidiospores. On the contrary, the strain did not mate with JEC20 (*MAT****a*** *D*) or with IUM 96-2828 (*MAT****a*** *A*).

The genotypic and phenotypic characteristics of the fungus were then compared with those of serotype A (*MAT****a*** and *MATα*) reference strains. The mating type was analyzed by using PCR amplification of *MF****a***, *MFα* genes, and *STE20****a*-** and *STE20α-*specific genes for serotype A and serotype D. PCR reaction was performed as previously reported (*4*). The amplification product showed that IUM 99-3617, like IUM 96-2828, contains only serotype A *STE20****a*** and *MF****a*** genes.

To further confirm that IUM 99-3617 was *MAT****a*** in mating type, *MF****a*** and *STE 20****a*** genes were sequenced by an ABI PRISM 310 automatic sequencer using Big Dye Terminator (Applied Biosystems, Monza, Italy) and the primers, forward and reverse strands previously reported (*4*). The sequences were then aligned with the reported sequences of IUM 96-2828 (*2*,*8*), the Tanzanian isolate 295.1 (*1*), H 99, and the congenic JEC 20 and JEC 21 strains. The IUM 99-3617 sequences were found to be identical to those of IUM 96-2828 and of the Tanzanian isolate 295.1. The *MF****a***A and the *STE20****a***A sequence of IUM 99-3617 have been submitted to GenBank database (www.ncbi.nlm.gov/Bankit/nhpbankit.cgi) under accession number AY182035 and AY182036, respectively.

Virulence studies in the mouse model demonstrate that, like IUM 96-2828, the strain is significantly less virulent than H99. The latter strain caused 100% deaths day 29, while IUM 99-3617 took until day 60 to kill 60% of mice (unpub. data). No difference was observed among the three serotype A strains when virulence factors such as capsule, melanin, phospholipase activity, and ability to grow at 37°C were tested.

The MAT***a*** of *C. neoformans* serotype A was long regarded as extinct or as existing in an undiscovered ecologic niche until the recent finding of the clinical and the environmental isolate (*1*,*2*). The existence of MAT***a*** in nature is also supported by recent studies designed to established the origin of the serotype AD strains (*4*,*5*). These studies demonstrated that AD strains were diploid or aneuploid hybrids derived from a fusion of serotype A and D parents and that several of them were harboring a serotype A MAT***a*** locus. These hybrid strains have been found fairly often in Europe (*9*,*10*).

The finding of this isolate provides evidence of the pathogenic role of this rare mating type, emphasizes the critical function of molecular genetic tools in the characterization of *C. neoformans* populations, and represents an advance in knowledge of this fungal species whose genome is undergoing identification by a worldwide research team.
